# Effects of Urban and Rural Resident Basic Medical Insurance on Healthcare Utilization Inequality in China

**DOI:** 10.3389/ijph.2023.1605521

**Published:** 2023-02-16

**Authors:** Liangwen Zhang, Rui Chen, Ya Fang

**Affiliations:** State Key Laboratory of Molecular Vaccinology and Molecular Diagnostics, School of Public Health, Xiamen University, Xiamen, China

**Keywords:** China, health insurance, difference-in-difference, healthcare utilization, inequality

## Abstract

**Objectives:** This study aims to evaluate the effects of Urban and Rural Resident Basic Medical Insurance (URRBMI) integration on healthcare utilization and explore the contribution of URRBMI to healthcare utilization inequality among middle-aged and older adults.

**Methods:** Using data from the China Health and Retirement Longitudinal Study (CHARLS) 2011–2018. The difference-in-difference model, concentration index (CI), and decomposition method were adopted.

**Results:** The results suggested that the probability of outpatient visits and the number of outpatient visits had decreased by 18.2% and 10.0% respectively, and the number of inpatient visits had increased by 3.6%. However, URRBMI had an insignificant effect on the probability of inpatient visits. A pro-poor inequality for the treatment group was observed. The decomposition revealed that the URRBMI contributed to the pro-poor inequality in healthcare utilization.

**Conclusion:** The findings suggest that URRBMI integration has decreased outpatient care utilization and improved the number of inpatient visits. While the URRBMI has improved healthcare utilization inequality, some challenges still exist. Comprehensive measures should be taken in the future.

## Introduction

In 2005, the WHO member states committed to achieving the goal of universal health coverage (UHC), to guarantee all people have equitable access to healthcare without the risk of financial ruin (1). An increasing number of developing countries have taken different measures to achieve UHC (2). Since the late 1990’s, China has launched a series of healthcare reforms and has established three main schemes: The Urban Employee Basic Medical Insurance (UEBMI), the New Cooperative Medical Scheme (NCMS), and the Urban Resident Basic Medical Insurance (URBMI). UEBMI was established in 1998 to provide medical insurance for urban workers and retirees in the formal sector. NCMS was established in 2003, in which rural residents voluntarily choose to participate. China began piloting the URBMI system in 2007 to address the issue of medical coverage for informally employed residents, jobless residents, older adults, and children in urban areas without UEBMI. In 2011, the percentages of urban and rural residents who had health insurance protection increased to 89% and 97.5%, respectively (3, 4). A brief introduction to the health insurance schemes in China was shown in Supplementary Table S1.

Despite significant progress in the healthcare system, the gap between NCMS and URBMI was growing. First, the urban-rural segmentation of health insurance had become an important factor in healthcare utilization inequality in rural areas (5). Second, the URBMI was implemented at the city level and the NCMS was implemented at the county level. The lower administration level of NCMS undermined the risk-sharing of health insurance (6). Gradually, reducing inequalities has become a growing concern of the public, and has been widely recognized as a major objective of health policies in China (7). To provide better healthcare services and financial protection for rural residents, some provinces and municipalities (i.e., Chongqing, Tianjin, Guangdong, Shandong, and Zhejiang) made a series of attempts to merge NCMS and URBMI from 2008 to 2015. In 2016, the Chinese government officially decided to merge NCMS and URBMI nationwide and established Urban and Rural Resident Basic Medical Insurance (URRBMI) with some specific guidelines. URRBMI is intended to cover urban residents, who are not eligible under UEBMI, and all rural residents. The total premiums of URRBMI are higher than NCMS and URBMI, and the population covered is substantially larger. This suggests that the URRBMI scheme could better perform the coverage function of the healthcare system for participants, and improve risk-sharing capacity. By enrolling in URRBMI, rural residents could receive the same level of reimbursement for medicines and healthcare services as urban residents and could enjoy a wider choice of facilities (8).

One of the target groups of URRBMI is older adults, a population with higher medical needs than the overall population, which poses challenges to URRBMI (9, 10). China has the largest population in the world and is aging rapidly. By the end of 2020, individuals aged 60 and above reached 264 million in China, accounting for 18.7% of the total population (11). The tendency of aging would result in increasing demands on health services and increasing the financial risk of health insurance funds. However, it remains unclear how URRBMI integration affects healthcare utilization among middle-aged and older adults in China.

The link between health insurance and healthcare utilization has been studied in a large body of literature. For some countries and regions that have implemented universal health insurance, such as Japan, South Korea, Thailand, Taiwan, and most European countries, the expansion of health insurance coverage has significantly improved the healthcare utilization of the population (12–15). A growing body of research showed that the effects of NCMS and URBMI had been generally positive, improving healthcare utilization, promoting equity in health financing, and decreasing out-of-pocket payment (16–18). Existing evidence on the effects of URRBMI integration is mixed. Liu et al. (19) found that the healthcare options and quality for rural residents were improved and medical expenses were significantly reduced after the implementation of URRBMI. Ma (20) reported that the implementation of URRBMI significantly increased healthcare utilization for rural residents, while Zhou et al. (8) found no impact on the likelihood of hospital admission among rural residents, due to differences in the data sources and methods used. Several studies have investigated inequality in URRBMI. Li et al. (21) revealed that the integration of URRBMI could improve equity in healthcare utilization. Ren et al. (22) provided evidence of benefit equity for outpatient care with the integration of URRBMI.

Overall, research on URRBMI has gradually increased in recent years, regarding medical expenses, healthcare utilization, catastrophic health expenditure, health outcomes, and financial protection (5, 8, 19, 21, 23, 24). However, most of the above literature used data before 2016 (the pilot phase of URRBMI), and there is little evidence of the effect of URRBMI after 2016 (the formal integration phase of URRBMI). After the integration of URRBMI nationwide, it is critical to investigate the effect of this policy on healthcare utilization and its inequality. Therefore, we used more recent data (up to 2018) to assess the effect of URRBMI integration after 2016, which could update the estimates in the previous literature. In this study, we aim to evaluate the effects of URRBMI integration on healthcare utilization, calculate the concentration index (CI) and decompose the CI to learn the contribution of URRBMI to the total inequality. The findings will provide references for the development of the URRBMI scheme, and lessons for other countries to achieve UHC.

## Methods

### Data Sources

The data for this study were derived from the China Health and Retirement Longitudinal Study (CHARLS), which is conducted by the National Development Research Institute of Peking University. The CHARLS was a nationally representative longitudinal survey targeting Chinese community-dwelling individuals aged 45 years and older along with their spouses. The CHARLS national baseline survey used multistage stratified probability-proportionate-to-size sampling to cover 28 provinces, 150 countries/districts, and 450 villages/urban communities, involving 17,708 individuals in 10,257 households (25). Using a four-stage and well-established sample design, CHARLS provides comprehensive information about demographics, health, insurance status, work and retirement, income and consumption, assets, as well as community-level information. In this paper, the panel data comprising from CHARLS 2011, 2013, 2015, and 2018 are selected as the research samples.

The CHARLS data allows us to identify whether sample individuals live in a province/municipality implementing the URRBMI. In this study, the treatment and control groups were determined according to whether the URRBMI was implemented in each province. We defined treatment provinces as those provinces which adopted the integration from January 2016 to December 2017 (Supplementary Table S2 presents the timeline of URRBMI integration in China). Five provinces and municipalities had an early integration of the URRBMI before 2016, which may lead to an underestimation of the URRBMI effects (26). Thus, we excluded these provinces and municipalities from the treatment group. The control provinces are those provinces that were integrated after 2017. The pre-integration period is assigned to the years 2011–2017 whereas the post-integration period is 2018.

We used 2011 survey data as the baseline, with 17,708 participants included in the study. 5,774 participants dropped out in the three waves from 2013 to 2018. We excluded participants if they lived in the five provinces and municipalities which implemented the URRBMI before 2016. Participants who were insured by other medical insurance (e.g., UEBMI or supplemental insurance) were excluded. In addition, participants under 45 years old and with abnormal or missing variables (e.g., gender, age, marital status, and self-reported health) were also excluded. The final sample of 6,204 participants with 5,438 in the treatment group and 766 in the control group (Supplementary Figure S1).

### Variables

The implementation of URRBMI is involved as an essential independent variable. The implementation of URRBMI = 1 means that the group is a treatment group, and the year is the post-integration period; otherwise, the implementation of URRBMI = 0.

The main dependent variables pertain to individuals’ healthcare utilization. Following the extant literature on the determinants of healthcare utilization in China using the CHARLS survey (27, 28), we constructed four dependent variables: 1) whether the individual had any outpatient visit last month; 2) whether the individual received any inpatient care last year; 3) the number of outpatient visits during last month; and 4) the number of inpatient visits during last year.

In light of existing literature (29–31), we used an adjusted Anderson Health Services Utilization Model as our starting point to select controlled variables, which might influence healthcare utilization. Three types of variables were controlled in the empirical analysis: the predisposing characteristics, including age, gender, marital status, and education status; the enabling resources, including residence and annual personal income; and the need factors, including chronic disease and self-reported health status. Supplementary Table S3 offers a list of summary statistics and a brief description of our variables.

## Statistical Analysis

### Difference-in-difference

The difference-in-difference (DID) approach is key to evaluating interventions to inform health policymakers and future policies. We employed the DID model to identify the impact of URRBMI on healthcare utilization.

$$Y_{it}=\beta_{0}+\beta_{1}URRBMI_{it}+\gamma X_{it}+\lambda_{j}+\eta_{t}+\varepsilon_{it}$$ (1)

In Eq 1, $Y_{it}$ is the dependent variable standing for healthcare utilization of participant *i* at time *t*. The coefficient $\beta_{1}$ is the key parameter of interest, which captures the effect of URRBMI on healthcare utilization. The key independent variable is $URRBMI_{it}$ , which is the interaction term of *Treat*_*i*_ and *Time*_*t*_. It equals one if participants come from a province where URRBMI is active in the post-integration period. Otherwise, it equals zero. $X_{it}$ represents a set of individual covariates, $\lambda_{j}$ represents the province fixed effect, $\eta_{t}$ represents the time fixed effects, and $\varepsilon_{it}$ is the error term. The single terms of the interaction (i.e., *Treat*_*i*_ and *Time*_*t*_) are absorbed in the fixed effects. Linear regression and logit models were estimated for continuous and binary outcomes, respectively (22).

### Concentration Index and Decomposition

The concentration index quantifies the degree of socioeconomic-related inequality in healthcare utilization (32). The index is bounded between −1 and 1, 0 denotes perfect equality, whereas a positive (negative) value indicates that a healthcare variable is more concentrated among the rich (the poor) middle-aged and older adults (7). The formula for computing the CI is:

$$C=\frac{2}{\mu }cov\left(h,r\right)$$ (2)

*h* represents the healthcare utilization indicators, r represents the fractional rank of personal income, μ represents the mean of *h*, and *cov* is the covariance between the healthcare utilization variable and the fractional rank of personal income.

The CI can be decomposed into the contributions of individual factors to income-related inequality, in which each contribution is the product of the sensitivity concerning that factor and the degree of income-related inequality in that factor (33). The decomposition of the concentration index is applied to the OLS regression. The regression model is as follows:

$$y=\alpha +\sum_{k}\beta_{k}x_{k}+\varepsilon$$ (3)

Where *y* is the variable of healthcare utilization, $\beta_{k}$ is the marginal effect of each *x*, ε indicates the error term. The decomposition of CI could be specified as follows:

$$C=\sum_{k}\left(\beta_{k}{\bar{x}}_{k}/\mu \right)C_{k}+GC_{\varepsilon }/\mu$$ (4)

Where ${\bar{x}}_{k}$ is the mean of $x_{k}$ , μ is the mean of the dependent variable, $C_{k}$ is the concentration index for $x_{k}$ , $GC_{\varepsilon }$ represents the generalized CI for *ε*.

All statistical analyses were performed using Excel V.2019 and Stata V.14.

## Results

### Descriptive Analysis

Table 1 shows the variables and the descriptive statistics of the sample. Overall, most participants were female, married, rural residents, and educated to below primary school. In 2011, most of the participants were 45–59 years old. Most participants had fair self-reported health status but one or more chronic health issues. The average annual personal income in the treatment and control groups were 1,773.37 yuan and 2,673.53 yuan respectively. In 2018, most of the participants were aged 60 years or above and had fair self-reported health status. The descriptive statistics of the dependent variables were shown in Supplementary Tables S4, S5.

**TABLE 1:** Variable definitions and summary statistics (China, 2011–2018).

Variables	2011		2018	
	Treatment (*n* = 5,438)	Control (*n* = 766)	Treatment (*n* = 5,438)	Control (n = 766)
Age				
45–59 years	3,287 (60.45)	453 (59.14)	1,631 (29.99)	217 (28.33)
≥60 years	2,151 (39.55)	313 (40.86)	3,807 (70.01)	549 (71.67)
Gender				
Male	2,480 (45.61)	346 (45.17)	2,480 (45.61)	346 (45.17)
Female	2,958 (54.39)	420 (54.83)	2,958 (54.39)	420 (54.83)
Marital status				
Married	4,889 (89.90)	693 (90.47)	4,554 (83.74)	637 (83.16)
Otherwise	549 (10.10)	73 (9.53)	884 (16.26)	129 (16.84)
Education status				
Illiterate	1,491 (27.42)	209 (27.28)	1,490 (27.40)	186 (24.28)
Primary or below	2,371 (43.60)	335 (43.73)	2,501 (45.99)	367 (47.91)
Junior or above	1,576 (28.98)	222 (28.98)	1,447 (26.61)	213 (27.81)
Residence				
Urban	1,467 (26.98)	268 (34.99)	1,467 (26.98)	268 (34.99)
Rural	3,971 (73.02)	498 (65.01)	3,971 (73.02)	498 (65.01)
Annual personal income^a^	1,773.37 (6,038.39)	2,673.53 (6,693.61)	4,971.69 (11,186.09)	8,727.58 (15,390.25)
Chronic disease				
Yes	3,769 (69.31)	492 (64.23)	2,474 (45.49)	301 (39.30)
No	1,669 (30.69)	274 (35.77)	2,964 (54.51)	465 (60.70)
Self-reported health status				
Good	1,183 (21.75)	249 (32.51)	1,009 (18.55)	196 (25.59)
Fair	2,886 (53.07)	359 (46.87)	2,572 (47.30)	390 (50.91)
Poor	1,369 (25.17)	158 (20.63)	1,857 (34.15)	180 (23.50)

Note: Mean (SD) was conducted for continuous variables; n (%) was conducted for categorical variables.

^a^ The unit of the annual personal income is RMB, we took the logarithm for personal income before running a regression.

### Difference-in-difference Estimates

The effects of URRBMI on healthcare utilization are presented in Table 2. For outpatient care utilization, the probability of outpatient visits decreased by 18.2% (OR = 0.818, *p* < 0.05), and the number of inpatient visits decreased by 10.0% (coefficient = −0.100, *p* < 0.01). These results indicated that the URRBMI integration had negative and significant effects on the outpatient care utilization of middle-aged and older adults. In terms of inpatient care utilization, we found that the probability of inpatient visits was slightly increased, but the effect was statistically insignificant (OR = 1.003, *p* > 0.1). In contrast, the number of inpatient visits increased by 3.6% (coefficient = 0.036, *p* < 0.05), which meant that the integration of URRBMI had positive effects on the number of inpatient visits.

**TABLE 2:** Effects of URRBMI on healthcare utilization (China, 2011–2018).

	Probability of outpatient visits		Number of outpatient visits		Probability of inpatient visits		Number of inpatient visits	
	Odds ratios	95%CI	Coefficients	SE	Odds Ratios	95% CI	Coefficients	SE
URRBMI	0.818**	(0.671, 0.998)	−0.100***	(0.026)	1.003	(0.767, 1.313)	0.036**	(0.015)
Age (Ref. 45–59 years)	0.942	(0.868, 1.021)	−0.011	(0.014)	1.330***	(1.186, 1.492)	0.048***	(0.012)
Gender (Ref. Female)	0.821***	(0.734, 0.918)	−0.083***	(0.027)	0.989	(0.919, 1.064)	0.003	(0.008)
Marital status (Ref. Otherwise)	0.947	(0.856, 1.049)	0.003	(0.052)	0.873	(0.773, 0.985)	−0.028*	(0.016)
Education (Ref. Illiterate)								
Primary or below	1.042	(0.935, 1.162)	0.007	(0.020)	1.089*	(1.000, 1.187)	0.010	(0.013)
Junior or above	1.119*	(0.987, 1.269)	0.024	(0.019)	1.122*	(0.995, 1.265)	0.016	(0.012)
Residence (Ref. Rural)	0.903**	(0.825, 0.989)	−0.010	(0.022)	1.175***	(1.064, 1.298)	0.020*	(0.011)
Annual personal income	1.006	(0.998, 1.014)	0.001	(0.002)	0.992*	(0.982, 1.001)	−0.002*	(0.001)
Chronic disease (Ref. No)	1.745***	(1.592, 1.912)	0.170***	(0.021)	1.877***	(1.760, 2.001)	0.093***	(0.011)
Self−reported health status (Ref. Good)								
Fair	1.849***	(1.676, 2.040)	0.136***	(0.019)	1.533***	(1.369, 1.716)	0.034***	(0.005)
Poor	3.945***	(3.606, 4.316)	0.584***	(0.059)	3.980***	(3.301, 4.799)	0.293***	(0.020)
Time effect	Yes		Yes		Yes		Yes	
Province effect	Yes		Yes		Yes		Yes	
Constant	0.241	(0.196, 0.297)	0.171***	(0.045)	0.013***	(0.010, 0.018)	0.031	(0.024)
*R* ^2^/Pseudo *R* ^2^	0.067		0.046		0.088		0.064	

Note: Odds Ratios (ORs) are reported for logit models, whereas coefficients are reported for linear regression models. Significance levels *p < 0.1; **p < 0.05; ***p < 0.01. Robust standard errors are reported in brackets and clustered by provinces. All regressions control for time−fixed effect and province−fixed effect. Abbreviations: URRBMI, urban and rural resident basic medical insurance.

#### Heterogeneity Analysis

To further explore the heterogeneous effects of URRBMI integration on different subgroups, we performed two additional analyses. As shown in Supplementary Table S6, for urban residents, the integration of URRBMI had a negative and significant effect on the probability of outpatient visits (OR = 0.752, *p* < 0.05). For rural residents, the URRBMI integration was associated with a 12.2% (coefficient = −0.122, *p* < 0.01) decline in the number of outpatient visits. In addition, the introduction of URRBMI had insignificant effects on the inpatient care utilization for rural residents. We also test this implication by running a subgroup analysis across the age group. The results showed that the implementation of URRBMI not only had significant negative impacts on outpatient care utilization (*p* < 0.01) but also had positive effects on the number of inpatient visits (*p* < 0.05) for the older group. There was no significant effect of URRBMI on healthcare utilization for the middle-aged group.

### Measurement of Inequality in Healthcare Utilization

Table 3 shows the change in the concentration index in healthcare utilization. In the treatment group, the CI of outpatient visits probability and number respectively decreased from −0.0124 to −0.0335 and from −0.0235 to −0.0539, indicating that the pro-poor inequality and lower-income people utilized more outpatient services. The CI of the inpatient visits probability increased from −0.0449 to −0.0203. The CI of inpatient visits number rose slightly but remained negative. This indicates that lower-income people also utilized more inpatient services. Regarding the control group, the CI of outpatient and inpatient visits probability remained positive from 2011 to 2018, implying that there is a pro-rich inequality. The CI values of the number of outpatient and inpatient visits in 2018 were negative, implying that there is a pro-poor inequality.

**TABLE 3:** The change of concentration index in healthcare utilization among treatment and control groups (China, 2011–2018).

Variables	Treatment group		Control group	
	2011	2018	2011	2018
Probability of outpatient visits	−0.0124	−0.0335**	0.0195	0.0100
Number of outpatient visits	−0.0235***	−0.0539**	−0.0084	−0.0049
Probability of inpatient visits	−0.0449	−0.0203	0.0588	0.0169
Number of inpatient visits	−0.0483**	−0.0467**	0.0948	−0.0219

Note: Significance levels *p < 0.1; **p < 0.05; ***p < 0.01.

Table 4 presents the decomposition results of CI for healthcare utilization in 2018. The CI of URRBMI implementation was negative (CI = −0.0215), meaning that lower-income people were more likely to be covered by it. The contribution rates of URRBMI implementation to the concentration index in the number of outpatient and inpatient visits were 9.96% and 6.59% respectively. The results indicated that URRBMI implementation contributed to the pro-poor inequality in healthcare utilization. In addition, the CI of having chronic diseases, and poor health status were negative, which reveals that lower-income people were more likely to be unhealthy. The factors that contributed most to the inequality were poor health status, annual personal income, and the age of 60 years and above.

**TABLE 4:** Decomposition of Concentration Index for healthcare utilization in 2018 (China, 2011–2018).

Variables	Number of outpatient visits				Number of inpatient visits			
	Elasticity	CI	Con.	Percentage	Elasticity	CI	Con.	Percentage
URRBMI implementation								
Yes	0.2647	−0.0215	−0.0057	9.96	0.1565	−0.0215	−0.0034	6.59
No	Ref.				Ref.			
Age								
45–59 years	Ref.				Ref.			
≥60 years	−0.1235	0.0889	−0.0110	19.25	0.1427	0.0888	0.0127	−24.90
Gender								
Male	−0.0652	0.1172	−0.0076	13.39	0.0511	0.1172	0.0060	−11.76
Female	Ref.				Ref.			
Marital status								
Married	0.0074	0.0086	0.0001	−0.11	−0.1375	0.0086	−0.0012	2.33
Otherwise	Ref.				Ref.			
Education status								
Illiterate	Ref.				Ref.			
Primary or below	0.0803	−0.0052	−0.0004	0.73	−0.0088	−0.0052	−0.0000	−0.09
Junior or above	0.0449	0.1143	0.0051	−8.99	−0.0084	0.1143	0.0010	1.89
Residence								
Urban	−0.0235	0.1428	−0.0034	5.90	0.0578	0.1428	0.0083	−16.26
Rural	Ref.				Ref.			
Annual personal income	−0.0350	0.3860	−0.0135	23.69	−0.0501	0.3860	−0.0194	38.02
Chronic disease								
Yes	0.1656	−0.0090	−0.0015	2.62	0.2354	−0.0090	−0.0021	4.17
Otherwise	Ref.				Ref.			
Self−reported health status								
Good	Ref.				Ref.			
Fair	0.1641	−0.0199	0.0033	−5.74	0.0866	0.0199	0.0017	−3.39
Poor	0.4222	−0.0888	−0.0375	65.77	0.4750	−0.0888	−0.0422	82.87

Note: The decomposition method is applied to the linear regression model. Therefore, we only presented the decomposition results of the number of outpatient visits and inpatient visits. Elasticity refers to the degree that the concentration index of each explanatory variable affects the concentration index of outpatient care utilization. CI, refers to the concentration index of each explanatory variable. Con. signifies the extant outpatient care utilization inequality can be attributed to each explanatory variable. Percentage denotes the percentage of each explanatory variable’s contribution to healthcare utilization inequality. Ref. is the reference group. Abbreviations: URRBMI, urban and rural resident basic medical insurance.

#### Common Trend and Robustness Analysis

The most important premise of the DID model is the common trend assumption. The assumption states that changes in healthcare utilization would be the same in both treatment and control provinces in the pre-integration periods (34). Supplementary Figure S2 indicated that there were no differences in the pre-integration trends for healthcare utilization, and the assumption of parallel trends was generally satisfied.

To demonstrate the robustness of the results, we combined the propensity score matching and difference-in-difference (PSM-DID) methods. The combination of two methods to counter biases and the confounding of different sources, and the comparison of results, are encouraged in the literature (23). The results of the estimation of the PSM-DID method are shown in Supplementary Table S7, which are consistent with the DID results reported in Table 2, indicating that the results of the DID model were robust.

In addition, we conducted a placebo test by constructing a false treatment group. Supplementary Figure S3 reports the kernel density of estimated coefficients for the 500 randomly generated treatment groups and the distribution of the associated *p*-values. These results indicated that the empirical results are not severely biased due to randomness and any omitted variables.

## Discussion

In this study, we estimated the effect of the URRBMI integration on healthcare utilization among middle-aged and elderly people in China from 2011 to 2018 with DID model. We also explored the changes in the healthcare utilization inequality before and after URRBMI integration, and further estimated the contribution of the URRBMI scheme to the inequality degree. Therefore, there are three aspects of our study that should be discussed.

Firstly, the empirical findings show that the URRBMI integration reduced the probability of outpatient visits and the number of outpatient visits, which is different from the evidence of earlier studies (36, 37). Meanwhile, the URRBMI integration significantly increased the number of inpatient visits, which is consistent with the previous study. This might have something to do with the fact that inpatient care has higher reimbursement rates and more medical services than outpatient care (38). Existing research has shown that health insurance has little association with the out-of-pocket cost of outpatient services, and patients can only receive greater reimbursement through inpatient care (36, 39, 40). This suggests that patients would reduce their use of outpatient care in favor of more inpatient care (41). Huang (6) proved that increased reimbursement rates after URRBMI integration could be a primary mechanism driving higher utilization of inpatient care. On the other hand, increased reimbursement for inpatient care might persuade some patients who would have previously sought outpatient care to choose the more expensive option, possibly encouraged by hospitals seeking to increase revenues. However, such behavior would lead to excessive medical treatment and increase the cost of healthcare utilization. Therefore, improving the benefits structure for outpatient care is imperative and guiding residents to use medical service resources rationally.

Second, the heterogeneous effects of URRBMI integration on each subgroup were observed. Compared with urban residents, the impact of URRBMI integration on rural residents was only reflected in outpatient care utilization and had an insignificant impact on inpatient care utilization, which differs from the expected results. URRBMI has a higher reimbursement rate in inpatient care than NCMS, which should improve the inpatient care utilization rate of rural middle-aged and older people, as confirmed by previous research results (20). The possible explanation is that there still be a gap in the distribution of medical resources between rural and urban areas, especially high-quality medical resources and inpatient services. Thus, the URRBMI impact on rural residents is only reflected in outpatient care utilization. Meanwhile, the differences in data collection, study samples, and populations would also lead to different findings, so further research is needed to validate these findings (42). In addition, the URRBMI significantly impacted healthcare utilization in older adults, but not in the middle-aged population, which implied that healthcare utilization was closely related to age. There may be two reasons: one is that as the age increases, the diseases appear and health gets worse, and the other is that older residents are more sensitive to the change in reimbursement rates. The higher reimbursement rates of the URRBMI scheme help to release the demand for healthcare among older adults.

Third, this study also finds that all of the CI values in the treatment group were negative after the URRBMI integration, indicating that the lower-economic participants had more healthcare utilization than the higher-economic participants. The findings are inconsistent with the previous study. Wang et al. (43) found that the richest utilize more outpatient care in URRBMI. Besides the differences in data and methods used, the time scopes of the study may also account for these different findings (36). By decomposing the concentration index, we found that URRBMI integration contributed to the pro-poor inequality in healthcare utilization. The effect is in alignment with the original intention of the URRBMI scheme. Furthermore, it is worth noting that poor health status has the greatest contribution to the inequalities of outpatient and inpatient care, which is consistent with earlier findings (44). This further proves that personal health status is the first driving force for healthcare utilization (44). Therefore, the government should play the role of URRBMI for middle-aged and older residents with poor health status, adopting better means to guide medical choices and promoting health.

There are several limitations to this study. First, since the decision to URRBMI integration in each province was affected by many unobservable factors (such as the willingness of local leaders), it is hard to avoid the problem of endogeneity (43). Second, the information on healthcare utilization was self-reported, which may have led to a reporting bias, especially for older respondents. Third, the post-integration period is only available for 1 year in 2018. The URRBMI integration effects may have a time lag effect that is not significant in the short term, which requires further evaluation with more observation time.

### Conclusion

This study shows that URRBMI integration has decreased outpatient care utilization and has improved the number of inpatient visits. Furthermore, the integration affected healthcare utilization for older adults. The URRBMI contributed to the pro-poor inequality in healthcare utilization. However, our findings also shed light on several areas where challenges remain. Therefore, comprehensive measures, such as improving the benefits structure of URRBMI to cover more outpatient diseases, optimizing the allocation of medical resources, and aiding vulnerable groups, should be taken during the post-integration period. The government should play the role of URRBMI for residents and adopt better means to guide medical choices. Improving the URRBMI scheme would be an important part of healthcare reform in China. Meanwhile, the experience in China may have broad implications for other low and middle-income countries that aim to reduce healthcare utilization inequality and achieve the goal of UHC.
