# Supplementary material for: Effects of Urban and Rural Resident Basic Medical Insurance on Healthcare Utilization Inequality in China
Source: Int J Public Health. 2023 Feb 16;68:1605521. doi: 10.3389/ijph.2023.1605521 (PMC9977786; doi:10.3389/ijph.2023.1605521)
Supplement: Supplementary file 1 [file DataSheet1.docx]

**Supplementary**

**Table S1.** Introduction of health insurance schemes in China (China, 2011-2018).

| **Insurance** | **UEBMI** | **NCMS** | **URBMI** | **URRBMI** |
| --- | --- | --- | --- | --- |
| Implementation year | 1998 | 2003 | Piloted in 2007 and implemented in 2010 nationwide | Piloted in several provinces in 2008 and implemented in 2016 nationwide |
| Target population | Urban employees and retirees | Rural residents | Urban residents without formal employment | Urban and rural residents without formal employment |
| Principle of participation | Mandatory | Voluntary | Voluntary | Voluntary |
| Funding | Payroll tax (6% from employers, and 2% from employees) | Individual contribution, collective support, and government subsidies | Individual contributions and government subsidies | Individual contributions and government subsidies |
| Number of people insured | 354.31 million (In 2021) | 670.29 million (In 2015) | 376.89 million (In 2015) | 1.0086 billion (In 2021) |
| Risk-pooling | Municipal/City level | County level | Municipal/City level | Municipal/City level |
| Per capita financing (RMB) | — | 490.3 (In 2015) | — | 889 (In 2021) |
| Insurance benefits | Covered outpatient and inpatient care | Mainly covered inpatient care, supplemented with outpatient care for catastrophic diseases and generous outpatient care | Mainly covered inpatient care, supplemented with outpatient care for catastrophic diseases | Mainly covered inpatient care, supplemented with outpatient care with serious illness and generous outpatient care |
| Inpatient reimbursement rates | 84.4% (In 2021) | — | — | 69.3% (In 2021) |

Note: Data are from the National Health Statistics Annual Report; The national statistical bulletin on the development of basic medical insurance.

Abbreviations: NCMS, the New Cooperative Medical Scheme; URBMI, the Urban Resident Basic Medical Insurance; URRBMI, Urban and Rural Resident Basic Medical Insurance; UEBMI, Urban Employee Basic Medical Insurance.

**Text S1.**

In 2016, the Chinese government officially issued “Opinions of the State Council on Integrating the Medical Insurance System for Urban and Rural Residents” (the “Opinions”) and decided to merge NCMS and URBMI nationwide and established Urban and Rural Resident Basic Medical Insurance (URRBMI). To achieve this objective, the “Opinions” provided guidance on 6 key areas, which included integrating the coverage of the medical insurance system, the fund-raising policies, the benefits, the health insurance directory, and the management of funds and selected agencies.

Actually, the implementation details are usually decided at a local level. In 2022, the national funding standard for URRBMI is 960 yuan per capita, of which 350 yuan is paid by individuals and 610 yuan is subsidized by the government. The actual funding standard of each province /municipality would be adjusted and formulated according to the local economic development level and demographic structure on the basis of meeting the national standard. For example, in Beijing, the funding standard for residents aged 19-59 is 3,665 yuan, of which the individual payment is 885 yuan and the financial subsidy is 2,780 yuan, which is much higher than the national standard. The funding standards in Fujian Province and Henan Province are set in accordance with national standards.

**Table S2.** Description of the URRBMI implementation in China and classification of treatment group and control group (China, 2011-2018).

| **Provinces** /**Municipalities** | **Time for implementation** |
| --- | --- |
| **Treatment group** | |
| Qinghai | June 2016 |
| Fujian | 2016 |
| Hebei | January 2017 |
| inner Mongolia | January 2017 |
| Shanghai | January 2016 |
| Hubei | June 2017 |
| Xinjiang | June 2017 |
| Jiangxi | 2017 |
| Shanxi | January 2017 |
| Anhui | 2017 |
| Henan | January 2017 |
| Hunan | January 2017 |
| Yunnan | January 2017 |
| Guangxi | January 2017 |
| Sichuan | January 2017 |
| Gansu | January 2017 |
| Jilin | January 2017 |
| Shaanxi | January 2017 |
| **Control group** | |
| Jiangsu | January 2018 |
| Heilongjiang | January 1^st,^ 2018 |
| Guizhou | January 2018 |
| Beijing | January 2018 |
| Liaoning | January 2020 |
| **Group of implemented URRBMI before 2016** | |
| Chongqing | 2009 |
| Tianjin | January 2010 |
| Guangdong | October 2012 |
| Shandong | September 2014 |
| Zhejiang | December 2014 |

Note: The CHARLS national survey was performed from July to August and reported inpatient utilization in the last year. Therefore, we used the one-year lagged adoption of URRBMI.

Data resource: The official website of the government of each province in China.

**
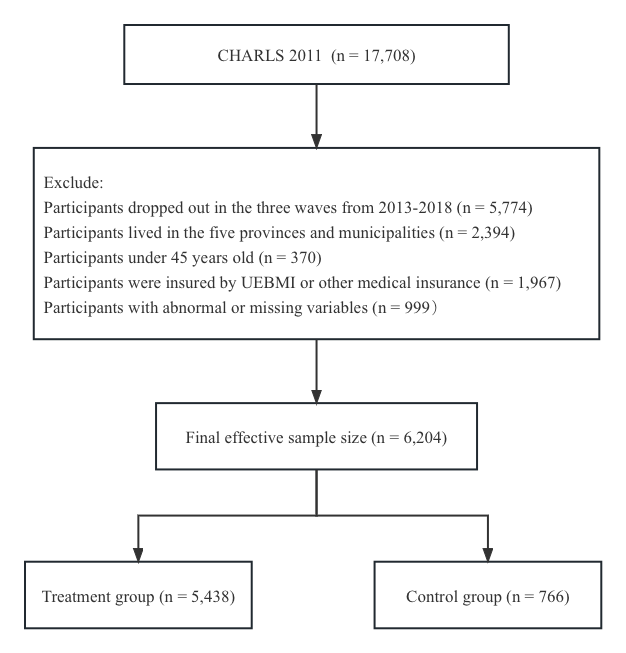
**

**Figure S1.** Study flow chart of sample selection (China, 2011-2018).

Note: We treated this longitudinal survey as panel data. The final sample of 24,816 observations from the 6204 participants.

**Table S3.** Definition and coding of variables (China, 2011-2018).

| **Variables** | **Definition** |
| --- | --- |
| **Dependent variables** | |
| Probability of outpatient visits | No = 0; Yes = 1 |
| Number of outpatient visits | Continuous variables |
| Probability of outpatient visits | No = 0; Yes = 1 |
| Number of inpatient visits | Continuous variables |
| **Control variables** | |
| **Predisposing characteristics** | |
| Gender | Female = 0; Male = 1 |
| Age | 45-59 years = 0; ≥60 years = 1 |
| Marital status | Otherwise (separated, divorced, widowed, never married and marital status cohabitated) = 0; Married = 1; |
| Education | Illiterate = 0; Primary or below = 1; Junior or above = 2 |
| **Enabling resources** |  |
| Residence | Rural = 0; Urban = 1 |
| Annual personal income | Continuous variables |
| **Need factors** |  |
| Chronic | No = 0; At least has one chronic disease (e.g., hypertension, dyslipidemia, diabetes or high blood sugar); Yes = 1 |
| Self-reported health status | Good = 0; Fair = 1; Poor = 2 |

**Table S4.** Descriptive statistics of the dependent variables (China, 2011-2018).

| **Variables** | **Treatment group (n=5,438)** | |  | **Control group (n=766)** | |
| --- | --- | --- | --- | --- | --- |
|  | **n** | **%** |  | **n** | **%** |
| **Panel A: 2011** | | | | | |
| Number (percentage) of respondents making outpatient visits in the last month | | | | | |
|  | 1,179 | 21.68 |  | 101 | 13.19 |
| Number (percentage) of respondents making inpatient visits in the last month | | | | | |
|  | 442 | 8.13 |  | 46 | 6.01 |
| **Panel B: 2018** | | | | | |
| Number (percentage) of respondents making outpatient visits in the last month | | | | | |
|  | 949 | 17.45 |  | 95 | 12.40 |
| Number (percentage) of respondents making inpatient visits in the last month | | | | | |
|  | 1,054 | 19.38 |  | 110 | 14.36 |

**Table S5.** Descriptive statistics of the dependent variables (China, 2011-2018).

| **Variables** | **Treatment group (n=5,438)** | |  | **Control group (n=766)** | |
| --- | --- | --- | --- | --- | --- |
|  | **Mean** | **SD** |  | **Mean** | **SD** |
| **Panel A: 2011** | | | | | |
| Mean number of outpatient visits | | | | | |
|  | 0.467 | 1.399 |  | 0.210 | 0.676 |
| Mean number of inpatient visits | | | | | |
|  | 0.112 | 0.513 |  | 0.068 | 0.290 |
| **Panel B: 2018** | | | | | |
| Mean number of outpatient visits | | | | | |
|  | 0.396 | 1.420 |  | 0.225 | 0.846 |
| Mean number of inpatient visits | | | | | |
|  | 0.313 | 0.815 |  | 0.209 | 0.601 |

**Table S6.** Heterogeneity Analysis (China, 2011-2018).

|  | **Probability of Outpatient Visits** | |  | **Number of Outpatient Visits** | |  | **Probability of Inpatient Visits** | |  | **Number of Inpatient Visits** | |
| --- | --- | --- | --- | --- | --- | --- | --- | --- | --- | --- | --- |
|  | **Odds ratio** | **95%CI** |  | **Coefficients** | **SE** |  | **Odds ratio** | **95%CI** |  | **Coefficients** | **SE** |
| **Panel A: urban residents** | | | | | | | | | | | |
| URRBMI | 0.752** | (0.475, 1.193) |  | -0.054 | (0.046) |  | 0.842 | (0.521, 1.360) |  | 0.034 | (0.041) |
| Control variables | Yes | |  | Yes | |  | Yes | |  | Yes | |
| Time effect | Yes | |  | Yes | |  | Yes | |  | Yes | |
| Individual effect | Yes | |  | Yes | |  | Yes | |  | Yes | |
| R^2^/ Pseudo R^2^ | 0.071 | |  | 0.050 | |  | 0.098 | |  | 0.078 | |
| **Panel B: rural residents** | | | | | | | | | | | |
| URRBMI | 0.821 | (0.578, 1.166) |  | -0.122*** | (0.033) |  | 1.182 | (0.819, 1.707) |  | 0.039 | (0.025) |
| Control variables | Yes | |  | Yes | |  | Yes | |  | Yes | |
| Time effect | Yes | |  | Yes | |  | Yes | |  | Yes | |
| Individual effect | Yes | |  | Yes | |  | Yes | |  | Yes | |
| R^2^/ Pseudo R^2^ | 0.067 | |  | 0.045 | |  | 0.087 | |  | 0.062 | |
| **Panel C: 45-59 years old** | | | | | | | | | | | |
| URRBMI | 0.887 | (0.534, 1.472) |  | -0.031 | (0.050) |  | 0.869 | (0.496, 1.520) |  | 0.007 | (0.036) |
| Control variables | Yes | |  | Yes | |  | Yes | |  | Yes | |
| Time effect | Yes | |  | Yes | |  | Yes | |  | Yes | |
| Individual effect | Yes | |  | Yes | |  | Yes | |  | Yes | |
| R^2^/ Pseudo R^2^ | 0.0736 | |  | 0.051 | |  | 0.090 | |  | 0.058 | |
| **Panel D: 60 years old and above** | | | | | | | | | | | |
| URRBMI | 0.780*** | (0.533, 1.099) |  | -0.126*** | (0.036) |  | 1.166 | (0.823, 1.653) |  | 0.049** | (0.020) |
| Control variables | Yes | |  | Yes | |  | Yes | |  | Yes | |
| Time effect | Yes | |  | Yes | |  | Yes | |  | Yes | |
| Individual effect | Yes | |  | Yes | |  | Yes | |  | Yes | |
| R^2^/ Pseudo R^2^ | 0.065 | |  | 0.044 | |  | 0.077 | |  | 0.064 | |

Note: Odds ratios (ORs) are reported for logit models, whereas coefficients are reported for linear regression models. Significance levels **P* < 0.1; ***P* < 0.05; ****P* < 0.01. Robust standard errors are reported in brackets and clustered by provinces. Additional controls included the variables of gender, age, marital status, education status, personal income, chronic disease, and self-reported health status. All regressions control for time-fixed effect and province-fixed effect.

Abbreviations: URRBMI, Urban and Rural Resident Basic Medical Insurance.

**Table S7.** Robustness analysis-The effects of URRBMI integration on healthcare utilization using PSM-DID model (China, 2011-2018).

|  | **Probability of Outpatient Visits** | |  | **Number of Outpatient Visits** | |  | **Probability of Inpatient Visits** | |  | **Number of Inpatient Visits** | |
| --- | --- | --- | --- | --- | --- | --- | --- | --- | --- | --- | --- |
|  | **Odds Ratios** | **95%CI** |  | **Coefficients** | **SE** |  | **Odds Ratios** | **95%CI** |  | **Coefficients** | **SE** |
| URRBMI | 0.815** | (0.665, 1.000) |  | -0.101*** | (0.026) |  | 1.005 | (0.768, 1.315) |  | 0.036** | (0.015) |
| Age (Ref. 45-59 years) | 0.942 | (0.868, 1.021) |  | -0.011 | (0.014) |  | 1.330*** | (1.187, 1.490) |  | 0.047*** | (0.012) |
| Gender (Ref. Female) | 0.820*** | (0.733, 0.919) |  | -0.082*** | (0.027) |  | 0.989 | (0.920, 1.064) |  | 0.003 | (0.008) |
| Marital status (Ref. Otherwise) | 0.946 | (0.855, 1.047) |  | 0.006 | (0.051) |  | 0.872** | (0.770, 0.988) |  | -0.028* | (0.016) |
| Education (Ref. Illiterate) |  |  |  |  |  |  |  |  |  |  |  |
| Primary or below | 1.044 | (0.935, 1.166) |  | 0.007 | (0.021) |  | 1.088* | (0.997, 1.188) |  | 0.010 | (0.013) |
| Junior or above | 1.120* | (0.988, 1.270) |  | 0.021 | (0.018) |  | 1.121* | (0.993, 1.265) |  | 0.015 | (0.012) |
| Residence (Ref. Rural) | 0.903** | (0.825, 0.988) |  | -0.011 | (0.023) |  | 1.176*** | (1.064, 1.300) |  | 0.020* | (0.011) |
| Annual personal income | 1.006 | (0.998, 1.014) |  | 0.000 | (0.002) |  | 0.992* | (0.982, 1.001) |  | -0.002* | (0.001) |
| Chronic disease (Ref. No) | 1.744*** | (1.591, 1.912) |  | 0.170*** | (0.021) |  | 1.874*** | (1.757, 1.999) |  | 0.092*** | (0.011) |
| Self-reported health status (Ref. Good) |  |  |  |  |  |  |  |  |  |  |  |
| Fair | 1.852*** | (1.677, 2.045) |  | 0.136*** | (0.020) |  | 1.533*** | (1.367, 1,718) |  | 0.034*** | (0.006) |
| Poor | 3.951*** | (3.607, 4.328) |  | 0.583*** | (0.061) |  | 3.983*** | (3.291, 4.822) |  | 0.294*** | (0.020) |
| Time effect | Yes | |  | Yes | |  | Yes | |  | Yes | |
| Province effect | Yes | |  | Yes | |  | Yes | |  | Yes | |
| Constant | 0.241*** | (0.196, 0.296) |  | 0.170*** | (0.045) |  | 0.013*** | (0.010, 0.018) |  | 0.031 | (0.024) |
| R^2^/ Pseudo R^2^ | 0.067 | |  | 0.046 | |  | 0.088 | |  | 0.064 | |

Note: Odds Ratios (ORs) are reported for logit models, whereas coefficients are reported for linear regression models. Significance levels **P* < 0.1; ***P* < 0.05; ****P* < 0.01. Robust standard errors are reported in brackets and clustered by provinces. All regressions control for time-fixed effect and province-fixed effect.

Abbreviations: URRBMI, Urban and Rural Resident Basic Medical Insurance.


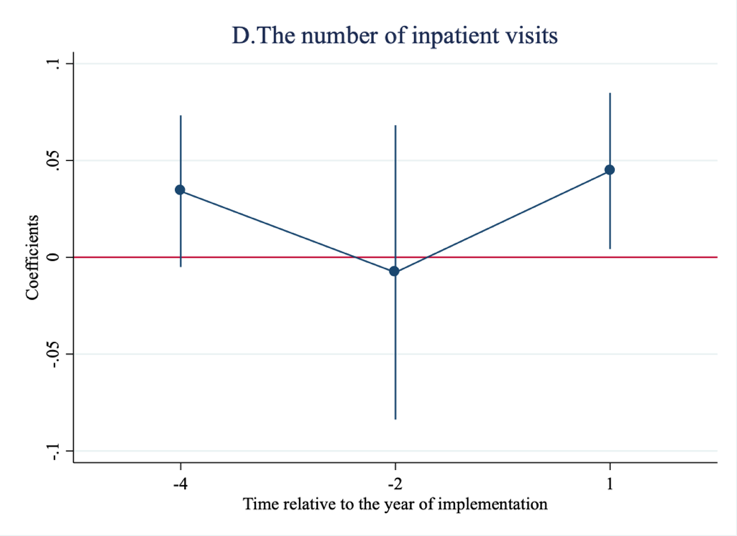

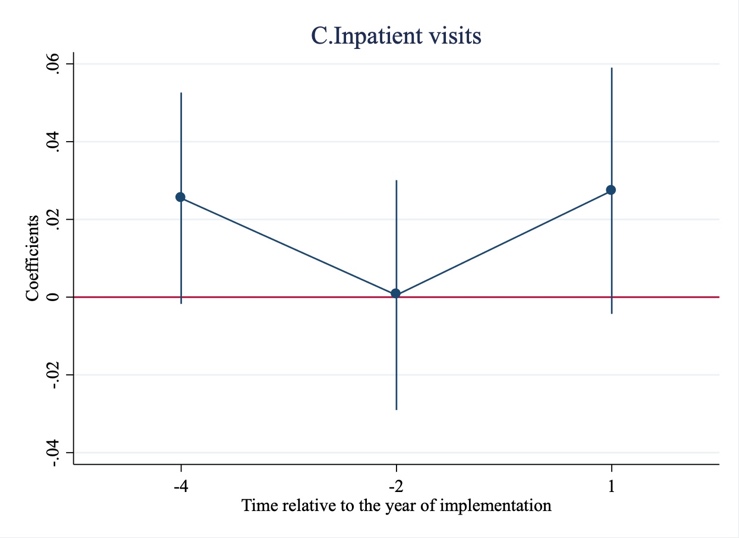

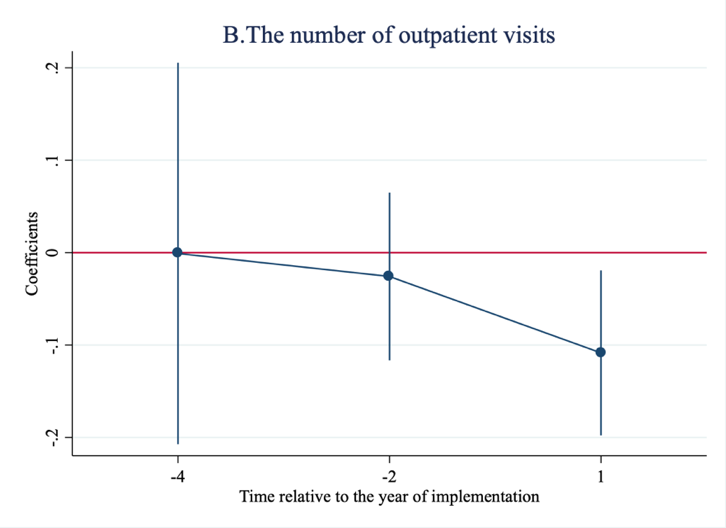

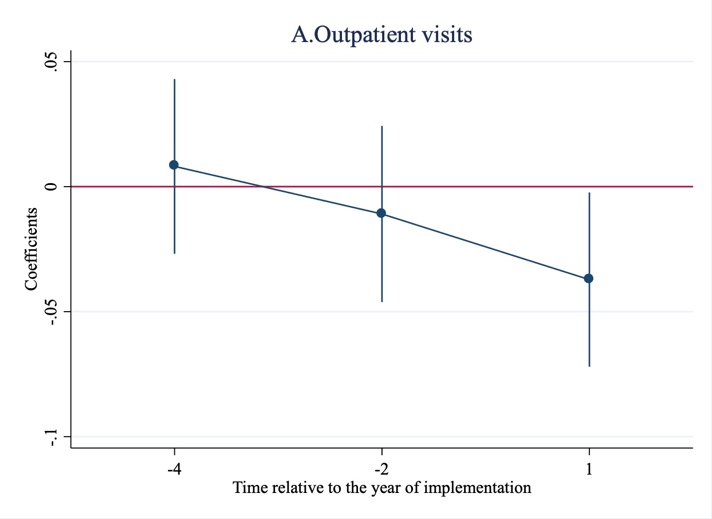


**Figure S2.** Common trend test (China, 2011-2018).


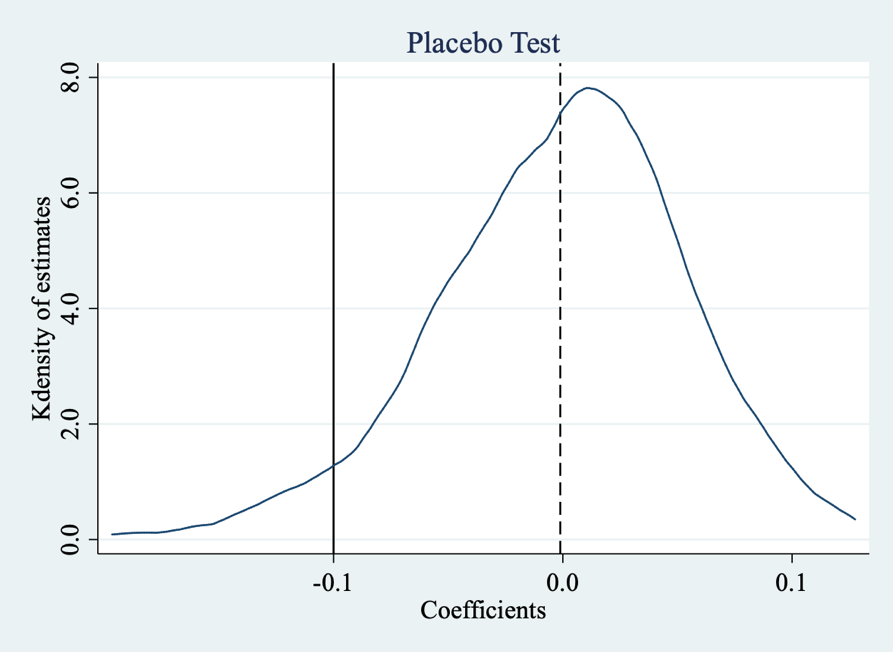


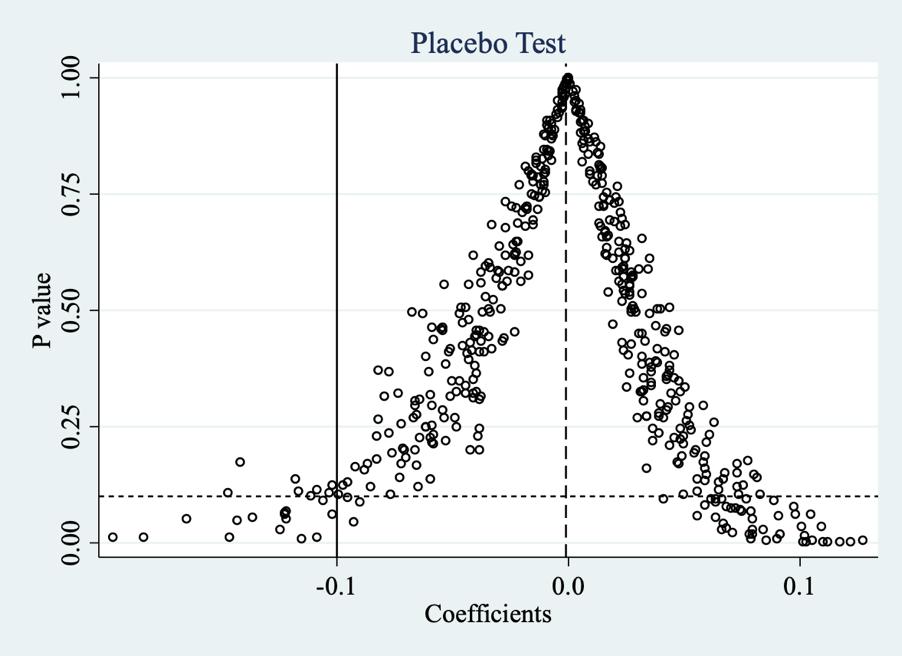


**Figure S3.** Placebo Test (China, 2011-2018).

Note: We took the placebo test for the number of outpatient visits.
